# Quinic acid protects against the development of Huntington’s disease in *Caenorhabditis elegans* model

**DOI:** 10.1186/s12906-024-04670-4

**Published:** 2024-10-28

**Authors:** Reem Hossam El Din, Sara Thabit

**Affiliations:** 1 Department of Pharmaceutical Microbiology, Faculty of Pharmacy and Biotechnology, German University in Cairo, New Cairo, 11835 Egypt; 2 Department of Pharmaceutical Biology, Faculty of Pharmacy and Biotechnology, German University in Cairo, New Cairo, 11835 Egypt

**Keywords:** *Caenorhabditis elegans*, PolyQ, SKN-1/Nrf2, Antioxidant

## Abstract

**Background:**

Quinic acid (QA), a cyclitol and cyclohexanecarboxylic acid, is a natural product that is present and can be isolated from edible herbals like tea, coffee and several fruits and vegetables. It was previously reported that QA exerted antioxidant and neuroprotective activity against dementia. However, it was not tested for its neuroprotective potential against Huntington’s disease (HD). Since aging related disorders are greatly linked to oxidative stress conditions, we focused on testing the oxidative stress resistant activity and protective effect of QA against the development of HD by using the multicellular *Caenorhabditis elegans* (*C. elegans*) worm model.

**Methods:**

Firstly, QA was tested for its oxidative stress resistant properties. In survival assay, wild type and mutant *skn-1* and *daf*-16 worms were exposed to oxidative stress conditions by using H_2_O_2_. Activation of SKN-1 pathway and expression of its downstream genes *gcs-1* and *gst-4* were also tested. Secondly, the effect of QA was evaluated on HD by testing its ability to decrease the formation of polyQ150 aggregates. Furthermore, its effect on the accumulation of polyglutamine (polyQ35 and polyQ40 aggregates) was tested.

**Results:**

Here we report that QA could improve the survival of *C. elegans* after exposure to oxidative stress caused by H_2_O_2_ while also exerting antioxidant effects through the activation of SKN-1/Nrf2 pathway. Moreover, QA could be a potential candidate to protect against HD due to its effects on decreasing the formation of polyQ150, polyQ35 and polyQ40 aggregates.

**Conclusions:**

This study highlights the importance of QA as a natural compound in defending against oxidative stress and the development of neurodegenerative diseases like HD.

**Supplementary Information:**

The online version contains supplementary material available at 10.1186/s12906-024-04670-4.

## Introduction

Dietary antioxidants and endogenous antioxidant enzymes aid in keeping reactive oxygen species (ROS) levels inside our bodies under control. An increase in ROS generation, due to their overproduction or a decrease in the antioxidant enzymes levels, leads to oxidative stress conditions. This eventually causes cellular damage, DNA mutations and several disorders [1, 2]. High levels of ROS can cause lipid and protein oxidation that can lead to aggregation and misfolding of structural proteins and, in consequence, the development of many neurodegenerative disorders [3]. Several studies have demonstrated the efficacy of nutraceuticals, spices and medicinal plants, having compounds with antioxidant effects, in protecting from several neurological, cardiovascular and metabolic disorders [1, 4].

Huntington’s disease (HD) is a progressive, dominantly inherited, neurodegenerative disorder. It occurs due to the pathological formation of an expanded polyglutamine (polyQ) sequence of the Huntingtin (Htn) protein in the N-terminal part leading to the formation of 35 or more CAG repeats. This disease is characterized by gradual reduction in cognitive abilities and motor skills, in addition to dementia. The length of the polyQ sequence is directly related to the onset and severity of the disease [5, 6]. Unfortunately, there is no treatment for HD available nowadays and patients are only treated symptomatically. Therefore, it is of great interest to search for drugs that could help by acting on different pathways or biochemical targets related to HD.

Natural products from plants, also termed phytochemicals, secondary metabolites or specialized metabolites, have always been interesting for nutrition and health for many years [7, 8]. They are also important in research related to oxidative stress, aging and neurodegenerative diseases [1, 9–11].Quinic acid (QA), 1,3,4,5-tetrahydroxycyclohexane-1-carboxylic acid, is a cyclic polyol with several hydroxyl groups. It is present, along with its derivatives like phenolic caffeoylquinic acids, in many nutritional products like coffee, tea, vegetables and fruits [12]. It is not made inside our bodies; however, its uptake in diet is very beneficial as it helps in the formation of essential amino acids like tryptophan, tyrosine and phenylalanine inside the gastrointestinal tract by microbes [13–15]. It was also previously documented that QA is a potential antioxidant compound by decreasing ROS intracellular levels in PC_12_ cells challenged with hydrogen peroxide (H_2_O_2_) [16]. Moreover, it was reported that it increased the survival of *Caenorhabditis elegans* (*C. elegans*) in vivo under both heat and oxidative stress conditions and was responsible for activating DAF-16 pathway and increasing the expression of SOD-3 in the *C. elegans* model [17]. Furthermore, it was capable of exerting neuroprotective effects in dementia induced by aluminum chloride in an in vivo rat model [18]. On the other hand, its effects on polyQ aggregate formation and development of HD was not reported.

The *C. elegans* nematode is an interesting simple multicellular in vivo model whose genome is entirely sequenced. It has homologs to approximately 67% of the genes linked to diseases in humans [19]. There are several transcription factors in this nematode responsible for the regulation of longevity, protein homeostasis and response to stress resistance. Therefore, it is an important model for the study of diseases related to proteotoxicity [20, 21]. It is a commonly used model organism to study the effects of ROS and antioxidants. Several mutants have been established in this model to allow studying ageing and several ageing related health conditions, including neurodegenerative diseases [22]. *C. elegans* SKiNhead-1 (SKN-1) transcription factor is orthologous to nuclear factor erythroid 2-related factor 2 (Nrf2) in humans. It is known for its participation in the maintenance of cellular redox homeostasis, xenobiotic defense, and its positive effects on life span and delay of age-associated proteotoxicity [23–26]. This points to the important role of the SKN-1/Nrf2 pathway in the process of aging and its related disorders.

The *C. elegans* model is used as a reliable model for studying the effect of several phytochemicals and extracts on the development of polyQ aggregates related to HD. This is due to the presence of *C. elegans* mutant strains expressing polyQ aggregates in their amphid sensilla (ASH) neurons as well as others in their muscles [27, 28].

To our knowledge, QA was neither tested for its effect on the activation of Ld-1 SKN-1 pathway nor against the development of polyQ aggregates as a marker for HD. Here, we used the *C. elegans* model to test the oxidative stress resistance induced by QA using the wild type, *skn-1* and *daf-16* null mutant nematodes. We also evaluated the potential involvement of the SKN-1/Nrf2 pathway in this process by investigating the nuclear localization of *skn-1* and the expression of the pathway’s downstream target genes: gamma-glutamyl cysteine synthetase (*gcs-1*) and glutathione S-transferase 4 (*gst-4*). The effect of QA on decreasing polyQ150, polyQ35 and polyQ40 aggregates development was tested using HA759, AM140 and AM141 strains respectively.

## Materials and methods

### *Caenorhabditis elegans* strains and reagents

Strains of *C. elegans* used in this study and *Escherichia coli* (*E. coli* OP50), came from IPMB, Heidelberg, Germany. The currently used strains are: Wild type N2, EU1 [*skn-1(zu67) IV/nT1(IV; V*)], CF1038 (*daf-16(mu86)I*), LD1 [*skn-1b/c::GFP + rol-6(su1006)*], LD1171 [(*gcs-1p: GFP* + *rol-6(su1006)*], CL2166 (*dvIs19[pAF15(gst-4::GFP::NLS)*]), AM140 (*rmIs132[unc-54p::Q35::YFP]*), AM141 (*rmls133[unc-54p::Q40::YFP]*) and HA759 (*rtIs11[osm-10p::GFP + osm10p::HtnQ150 + Dpy-20(+)]*). Nematode growth medium (NGM) plates, containing *E. coli* OP50 as a source of nutrition for the nematodes, were incubated at 20 °C. Synchronization of worms, in order to have a uniform aged population, was done with our established bleaching protocol utilizing 5% NaOCl and 5 M NaOH in a ratio of 3:1 [29]. All reagents used in this study were bought from Sigma-Aldrich, Germany. QA (98%) stock was prepared using sterile water and stored in -20 °C. H_2_O_2_ solution 30% (w/w) in H_2_O was used to make fresh preparations of 15 mM concentration directly before stress resistance assay. 5′-fluorodeoxyuridine (FUDR), 98%, was used to prevent reproduction in worms. Benzaldehyde (98%) was used as an odorant.

### Stress resistance assessment

This assay was performed to investigate the ability of QA to protect the worms from oxidative damage induced via the well-known oxidant, H_2_O_2_ [30]. Age-synchronized wild type nematodes (N2), EU1 (*skn-1* null mutants) and CF1038 (*daf-16* null mutants) were used in this assay to determine whether the SKN-1/Nrf2 pathway is involved in the activity of the tested compound or not. L1 stage worms of both strains were segregated into groups of 70–80 worms and grown in S-medium and *E. coli* OP50 (OD_600_ = 1.0) at 20 °C. The groups included are: untreated control group (receiving only water, the solvent used for dissolving QA, without the pro-oxidant H_2_O_2_), H_2_O_2_ treated group (ROS control), QA groups at doses of 50, 100 and 200 µg/ml [17]. All QA groups were preincubated for 72 h then subjected to a dose of 15 mM H_2_O_2_ for 3 h. After 3 h, dead and alive 2-days-old adult worms were counted and recorded as previously described [31]. All experiments were repeated three times and data was calculated as the mean ± SEM of the survival percentage. Comparison of results was performed via GraphPad prism (5.01) where significance was tested via one-way ANOVA and Bonferroni’s method (post hoc).

### SKN-1 localization assay

The LD1 transgenic strain, characterized by having SKN-1 coupled to green fluorescent protein (GFP), was used in this assay. L1 nematodes were divided and treated with QA (at concentrations of 50, 100 and 200 µg/ml). In addition, an untreated control group was used. Treatment groups were preincubated for 48 h in S-medium and *E. coli* OP50 at 20 °C. SKN-1::GFP distribution within the 1-day-old adult worms was visualized using fluorescent microscopy. About 15 worms were viewed and analyzed using constant exposure time at 20X magnification. An Axiostar Plus 37081 fluorescence microscope (Carl Zeiss) was utilized to visualize fluorescence inside the worms.

The assay was repeated three times and worms were sorted based on their SKN-1::GFP localization to cytosolic or nuclear. The percentage of worms with nuclear localization was assessed.

### GCS-1 expression assay

Synchronized L1 worms of the LD1171 strain, expressing the GFP reporter for *gcs-1*, were utilized in this assay. Worms were cultivated in S-medium and *E. coli* OP50 at 20 °C. They were sorted into four groups and treatment was carried out as described above. Afterwards, they were kept for 48 h at 20 °C. On reading day, worms in their 1st day of adulthood were placed on a glass slide and paralyzed using 10 mM sodium azide. Photos were taken for 10–15 worms using a 10X objective lens and constant time of exposure. The whole body was measured for its mean fluorescence intensity via ImageJ software version 1.48 (NIH, Bethesda, MD, USA). The experiment was repeated three times.

### GST-4 expression assay

L1 larvae of the CL2166 strain, expressing GST-4 coupled to GFP, were used in this assay. Nematodes were cultured in S-medium having *E. coli* OP50 at 20 °C. They were divided into four groups and treated as described above for GCS-1. Afterwards, they were kept for 72 h at 20 °C. On reading day, worms in their 2nd day of adulthood were placed on a glass slide and paralyzed using 10 mM sodium azide; approximately 10–15 worms were photographed using a 10X objective lens and constant exposure time. Mean fluorescence intensity was investigated in the worms’ bodies. The experiment was repeated three times.

## Neuronal viability assay

The HA759 strain was utilized to assess survival of neurons as described formerly. Worms from this strain express Htn-Q150 coupled to GFP in a couple of ASH sensory neurons found in the head region. These neurons become progressively dysfunctional over time. Worms suffering from neurodegeneration are those with only one fluorescent neuron instead of two [32, 33]. After synchronization, L1 worms were separated into two groups: control and 200 µg/ml QA and kept in S-medium and *E. coli* OP50 for 72 h. On reading day, 2-days adult worms were collected then placed on a glass slide and immobilized using 10 mM sodium azide. About 30 nematodes were viewed with a10X objective lens using constant exposure time. Data is presented by using the average of three different trials of experiments. The neuronal survival percentage was then calculated as follows:

$$\:\frac{\text{N}\text{u}\text{m}\text{b}\text{e}\text{r}\:\text{o}\text{f}\:\text{w}\text{o}\text{r}\text{m}\text{s}\:\text{s}\text{h}\text{o}\text{w}\text{i}\text{n}\text{g}\:\text{t}\text{w}\text{o}\:\text{f}\text{l}\text{u}\text{o}\text{r}\text{e}\text{s}\text{c}\text{e}\text{n}\text{t}\:\text{n}\text{e}\text{u}\text{r}\text{o}\text{n}\text{s}}{\text{T}\text{o}\text{t}\text{a}\text{l}\:\text{n}\text{u}\text{m}\text{b}\text{e}\text{r}\:\text{o}\text{f}\:\text{w}\text{o}\text{r}\text{m}\text{s}}*100$$

Data is expressed as a mean % ASH neuronal survival and results were compared using Student’s t-test.

### Chemotaxis assay

This assay was performed as previously mentioned to check the viability of ASH neurons in HA759 worms by testing their chemosensory behavior [34]. Two groups of L1 nematodes, 60–80 worms each, were used. Groups were sorted into a control and 200 µg/ml QA. After treatment, groups were left in a 20 °C incubator with S-medium and *E. coli* OP50 for 72 h. Afterwards, day 2 adult worms were transferred to specific 60 mm NGM chemotaxis plates. The plates were divided into two equal quadrants (X and Y). On each quadrant, a drop of 0.5 M sodium azide was added to the side to induce paralysis of worms in that quadrant. A drop of 0.1% benzaldehyde, as an odorant, was added to quadrant X and a drop of 99.8% ethanol was added to quadrant Y. Directly before performing the experiment, HA759 worms were rinsed three times with M9 buffer to take off any traces of *E. coli* OP50. Afterwards, adult worms were added to a spot in the center of each plate and left in 20 °C incubator. After 1 h, the number of worms was scored in each quadrant and chemotaxis index was computed according to the following equation:

Chemotaxis index (C.I) = number of worms in X - number of worms in Y / collective number in X + Y.

The assay was repeated three times and results were evaluated as mentioned previously in neuronal viability assay.

### Assessment of PolyQ35 clusters formation

AM140 worms, with polyQ35 coupled to YFP (polyQ35::YFP), were employed in this assay. This assay investigates the aggregation of polyQ35 protein in the muscles. Aggregation is age-dependent, usually starting at day 3 of adulthood when the protein starts converting from the soluble to the aggregated state accumulating in the muscles of the body wall [35]. Worms were divided into two groups, as prescribed above, via S-medium and *E. coli* OP50 in the 20 °C incubator. FUDR was added at a concentration of 160 µM to worms at the L4 stage to prevent progeny. Worms were assessed after 96 h and 120 h of treatment (3rd and 4th day of adulthood). They were collected on the reading day and analyzed by fluorescence microscopy as described above. Almost 30 worms/group were visualized and the number of polyQ35 clusters was counted. Experiments were repeated three times and results are expressed as mean of aggregate numbers.

### Assessment of PolyQ40 clusters formation

AM141 worms, with polyQ40 coupled to YFP (polyQ40::YFP), were used in this assay. The purpose of this assay is to investigate the formation of polyQ40 clusters. L1 worms were split into two groups, as previously mentioned, and kept in S-medium and *E. coli* OP50 in the 20 °C incubator. Worms were evaluated after 3 days of treatment. Worms, at the 2nd day of adulthood, were gathered and analyzed by the previously mentioned fluorescence microscopy method. About 30 worms/group were analyzed via counting the number of polyQ40 clusters present in body wall muscles. Experiments were repeated three times and results are expressed as mean of clusters numbers.

### Body bending assay

Age synchronized L1 larvae (AM140 and AM141strains) were used in the current assay for the assessment of the locomotive ability of the worms. Two groups were used in this assay, the untreated control and the QA 200 µg/ml group. FUDR was added at the L4 stage to block progeny. Readings were done on day 1, 5 and 10 of adulthood. Worms were washed with M9 buffer for a period of 120 s to recover. Locomotion was assessed using body bends count with a stereomicroscope, which was used for observation over a period of 30 s. A thrash is counted once a worm alters its bending direction from the middle part of its body [36]. Sixty worms were counted from each group and the experiment was repeated three times. Results are presented as an average number of body bends per thirty seconds.

## Results

### Effects of QA on oxidative stress resistance

**Fig. 1:**
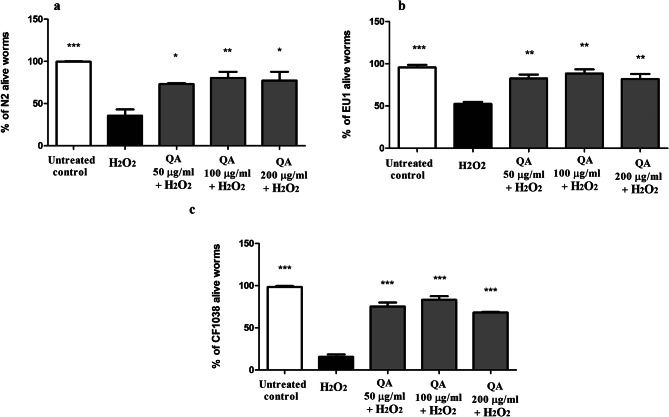
QA increases tolerance to oxidative stress caused by 15 mM H_2_O_2_. **a** N2 worms treated with QA 200 µg/ml showed 77.33 ± 10% survival compared to the H_2_O_2_ group, 35.67 ± 7% (*p < 0.05). **b** EU1 worms given QA 200 µg/ml demonstrated also a significant increase in survival, 81.77 ± 6%, in comparison with H_2_O_2_ group, 52.38 ± 2% (**p < 0.01). **c** CF1038 worms treated with QA 200 µg/ml resulted in a survival rate of 68.10 ± 0.7% compared to the H_2_O_2_ group (15.53 ± 3%) (***p < 0.001). Data represents the mean survival percent ± SEM of three different trials compared through one-way ANOVA then post-hoc Bonferroni technique

To test for the oxidative stress resistance potential of QA, QA-treated worms from N2, EU1 and CF1038 strains were challenged with 15 mM H_2_O_2_ for 3 h. N2 is the wild type strain having no mutations. EU1 is a null mutant *skn-1* strain that lacks SKN-1 transcription factor responsible for lifespan extension and response to oxidative stress. CF1038 is a *daf-16* null mutant. It is a short-lived strain that lacks DAF-16 transcription factor, an important regulator for several genes controlling stress response and longevity [37, 38]. The percentage of surviving worms pretreated with different doses of QA was significantly higher than that of the H_2_O_2_-treated group in all strains. Using N2 worms, QA-treated worms at all doses showed a significantly higher percentage of living worms compared to the H_2_O_2_-treated group. QA (200 µg/ml) resulted in a survival rate of 77.33 ± 10% (*p < 0.05) compared to the H_2_O_2_ group (35.67 ± 7%) (Fig. 1a). Moreover, all QA-treated groups of the EU1 strain showed significant increase in survival rates compared to the H_2_O_2_ group. QA (200 µg/ml) also showed a survival rate of 81.77 ± 6% (**p < 0.01) compared to the H_2_O_2_ group (52.38 ± 2%) (Fig. 1b). Finally, QA-treated groups of CF1038 strain also showed higher survivability compared to the H_2_O_2_ group. QA (200 µg/ml) resulted in a survival rate of 68.10 ± 0.7% (***p < 0.001) compared to the H_2_O_2_ group (15.53 ± 3%) (Fig. 1c). These results highlight that the oxidative stress resistance activity of QA might be mediated but is not strictly dependent on the activation of Ld-1 SKN-1 or DAF-16 pathways and other pathways might be involved in this effect.

### Effects of QA on SKN-1 localization

To further investigate whether or not the Ld-1SKN-1 pathway plays a role in the activity of QA, SKN-1 localization was examined in LD-1 worms. SKN-1 is considered as the orthologue of the mammalian Nrf-2 that is responsible for the regulation of several genes, both related and unrelated to stress conditions.

**Fig. 2:**
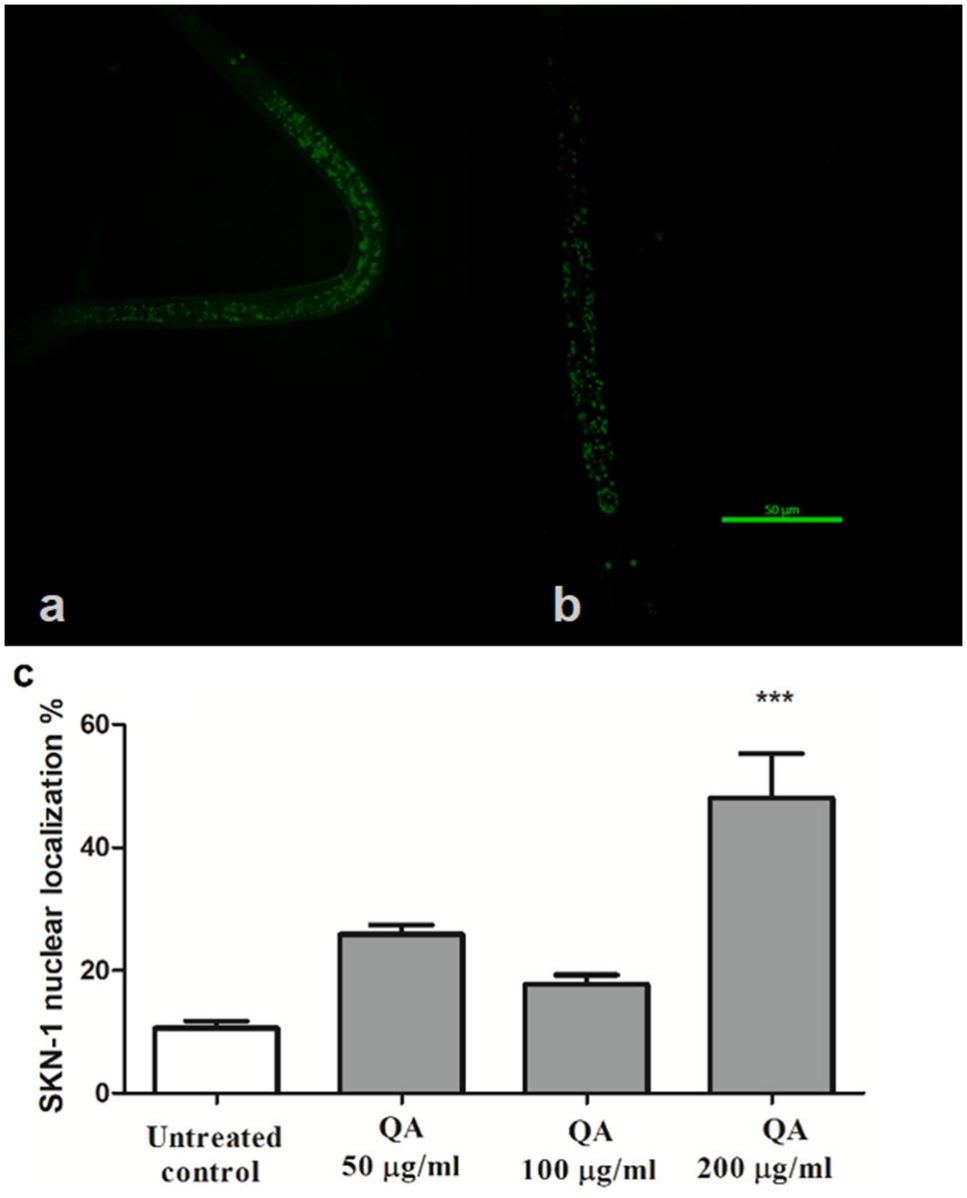
Effect of QA on SKN-1 translocation. **a** Image displaying cytoplasmic localization. **b** Image displaying nuclear localization. Scale bar = 50 μm. **c** Percentage of worms showing nuclear localization. QA 200 µg/ml enhanced SKN-1 nuclear localization (48.02 ± 7%) when compared to the untreated control (10.64 ± 1%) (***p < 0.001). Data denotes the average nuclear percentage ± SEM of three different trials compared through one-way ANOVA then post-hoc Bonferroni technique

Worms expressing a GFP labeled *skn-1* promoter were utilized in this assay. The highest dose of the drug (QA 200 µg/ml) led to a significant translocation of SKN-1 from the cytosol to the nucleus (48.02 ± 7%) in relation to the untreated control (10.64 ± 1%) (***p < 0.001) (Fig. 2).

### Effect of QA on GCS-1 expression

**Fig. 3:**
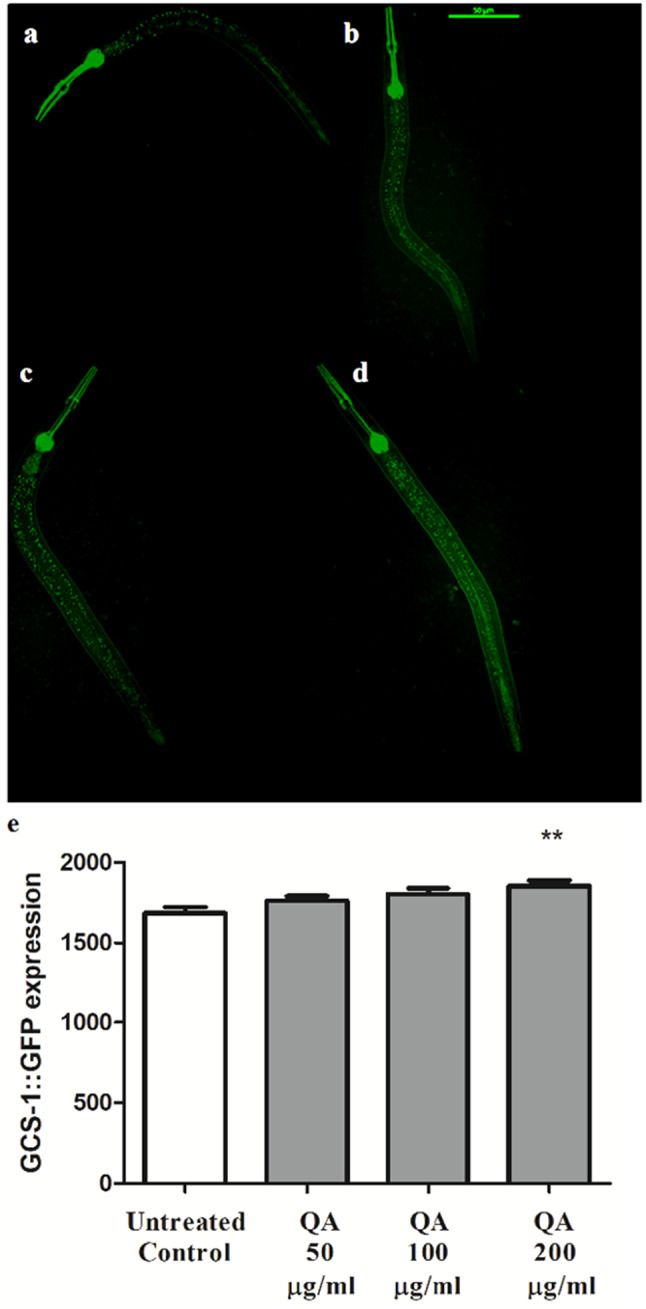
Effect of QA on the expression of GCS-1. **a** Untreated control. **b** QA 50 µg/ml. **c** QA 100 µg/ml. **d** QA 200 µg/ml. Scale bar = 50 μm. **e** QA 200 µg/ml enhanced the expression of *gcs-1* with 10% compared to the untreated control (**p < 0.01). Data is expressed as the mean of three experiments ± SEM; comparison is done through one-way ANOVA and post-hoc Bonferroni technique

Upon certain constitutive conditions or exposure to stress, SKN-1 accumulates inside the nucleus and activates its downstream genes: *gcs-1* and *gst-4*, which exert antioxidant activity. GCS-1 plays a role in the synthesis of glutathione (GSH) by catalyzing its first step. It is usually expressed in both the anterior and posterior intestine, pharynx and ASI chemosensory neurons of *C. elegans*. GCS-1 is important for the protection from oxidative stress and the promotion of longevity [39]. Results of this assay showed that QA 200 µg/ml increased the expression of *gcs-1* significantly by 10% in comparison to the control group, emphasizing the result of the previous assay that Ld-1 SKN-1 transcription factor is involved in the activity of QA (**p < 0.01) (Fig. 3).

### Effect of QA on GST-4 expression

GST-4 is a member of glutathione S-transferases family and it is the most studied form in *C. elegans* [39]. The expression of *gst-4*::GFP in worms, in response to different stressors, is mainly in the intestine [40, 41]. Increasing the expression of GST-4 leads to a rise in stress resistant properties of the worms against external stressors like juglone and paraquat [42].

**Fig. 4:**
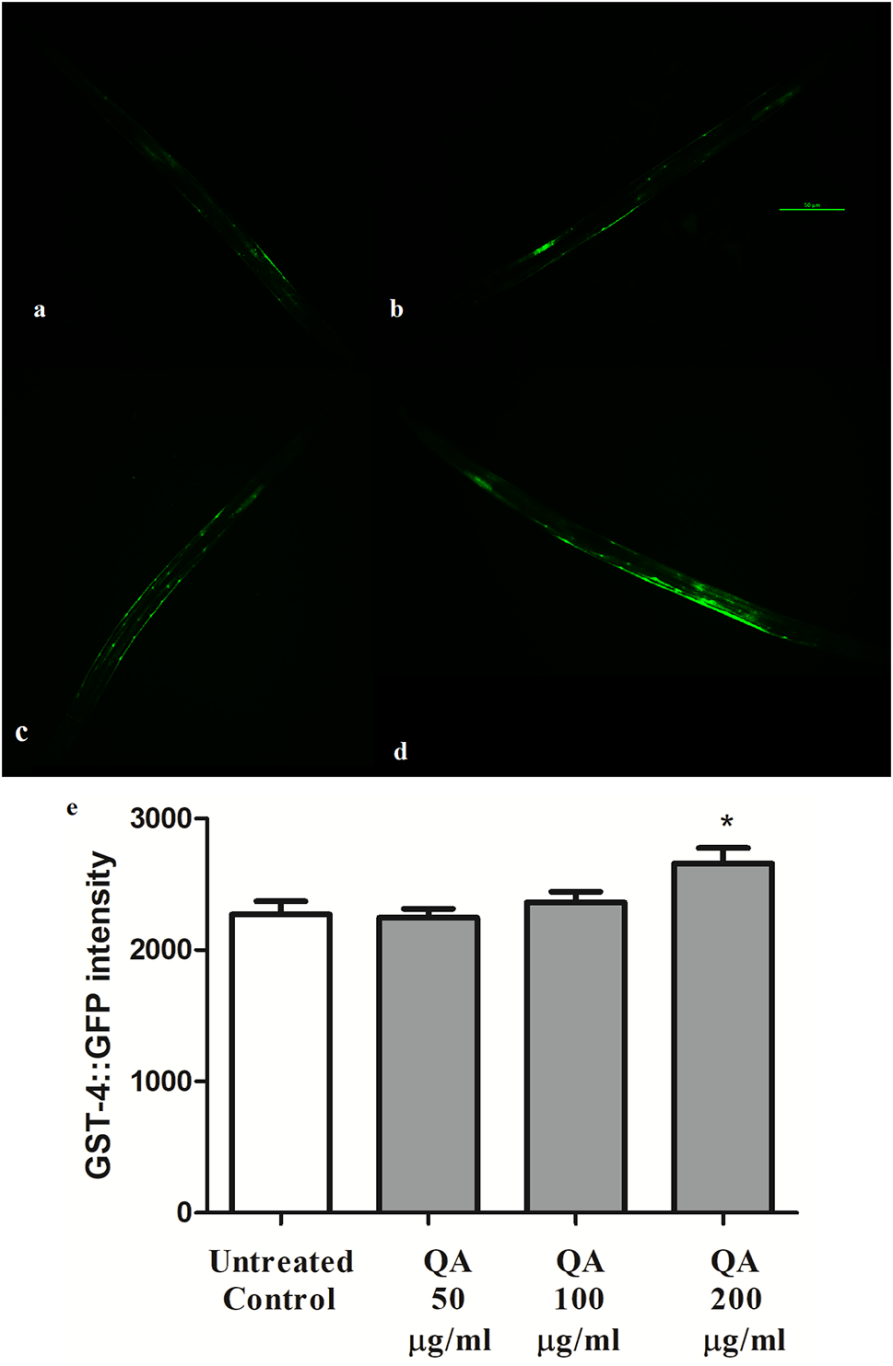
Effect of QA on GST-4 expression. **a** Untreated control. **b** QA 50 µg/ml. **c** QA 100 µg/ml. **d** QA 200 µg/ml. Scale bar = 50 μm. **e** QA 200 µg/ml increased the expression of *gst-4* with 17% in comparison with the untreated control (*p < 0.05). Data is expressed as a mean of three experiments ± SEM; comparison is done through one-way ANOVA and post-hoc Bonferroni technique

Results show that 200 µg/ml QA caused a significant rise in the GST-4 expression in comparison to the control group; emphasizing the result of the previous assays that Ld-1 SKN-1 transcription factor is involved in the activity of QA. An increase in fluorescence intensity with 17% relative to control was noticed (p* < 0.05) (Fig. 4).

### QA decreased polyQ150 aggregate formation

**Fig. 5:**
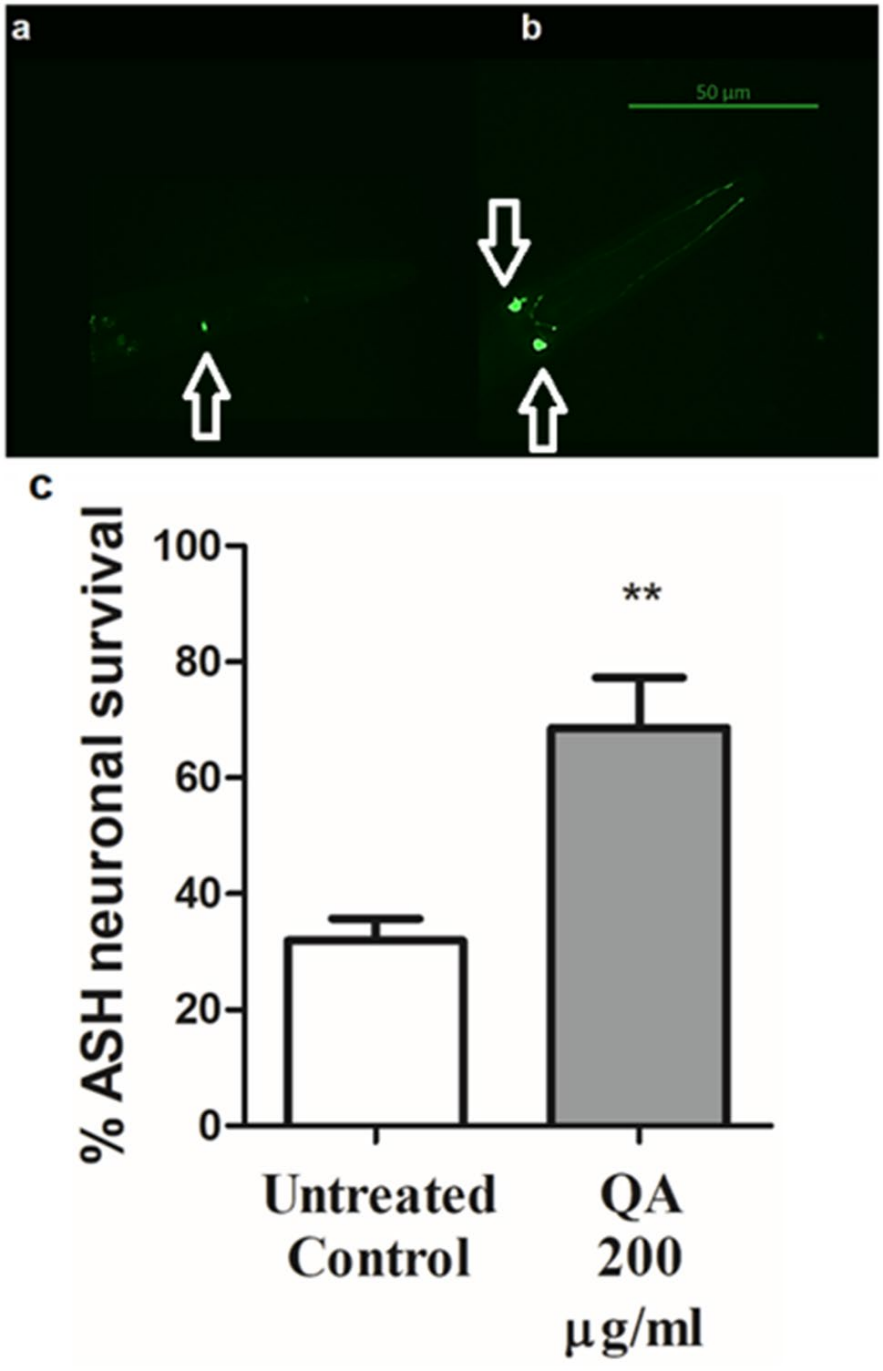
Impact of QA on ASH neuronal survival. **a** HA759 worm showing death in one of the ASH neurons. **b** HA759 worm with bilateral ASH neurons. Scale bar = 50 μm. **c** QA 200 µg/ml increased the percentage of ASH neuronal survival by 36% compared to the untreated control (**p < 0.01). Data is represented as a mean % ASH neuronal survival ± SEM and results comparison was performed using Student’s t-test

HA759 worms were used to analyze the protective effect of QA on the development of polyQ150 aggregates that lead to neurodegeneration of ASH neurons. QA (200 µg/ml) was chosen to be further tested based on the results of the SKN-1 pathway assays. Figure 5 shows a significant rise in ASH neuronal survival percentage in QA-treated worms versus the untreated control group by 36% (**p < 0.01).

**Fig. 6:**
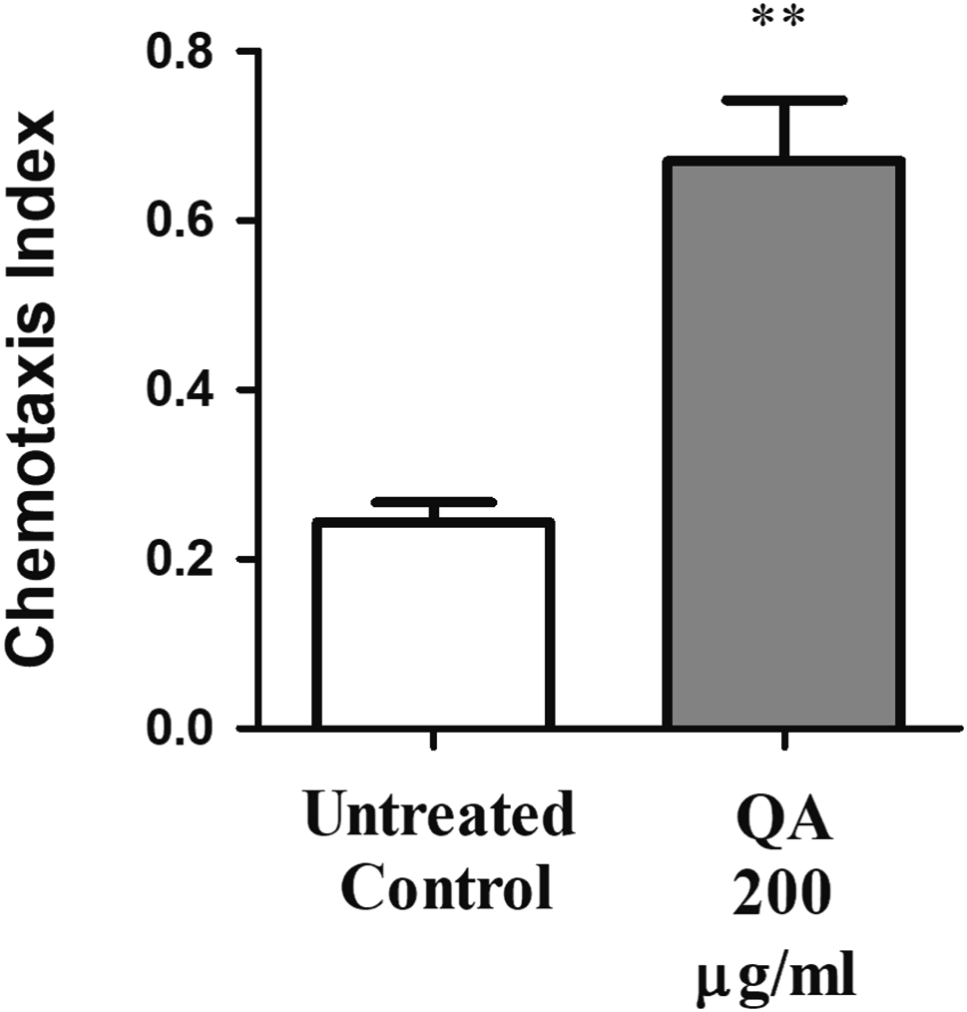
Assay for chemotaxis. QA 200 µg/ml resulted in elevation in chemotaxis index by 179% in comparison to the untreated control group (**p < 0.01). Results are shown as mean chemotaxis index ± SEM and data comparison was performed using Student’s t-test

ASH neurons in *C. elegans* play a key role in their sensory reactions towards odorants like benzaldehyde, which affect their chemotaxis behavior [43]. Since an accumulation of polyQ150 aggregates lead to defective ASH neuronal function, a chemotaxis assay was performed for further testing the protective ability of QA on ASH neurons. Treatment with 200 µg/ml QA increased the chemotaxis index significantly by 179% compared to the untreated control group (**p < 0.01) (Fig. 6). Together, results using HA759 worms show that QA was able to decrease polyQ150 aggregate formation in ASH neurons and protect from neurotoxicity.

### QA decreased polyQ35 and polyQ40 aggregate formation

**Fig. 7:**
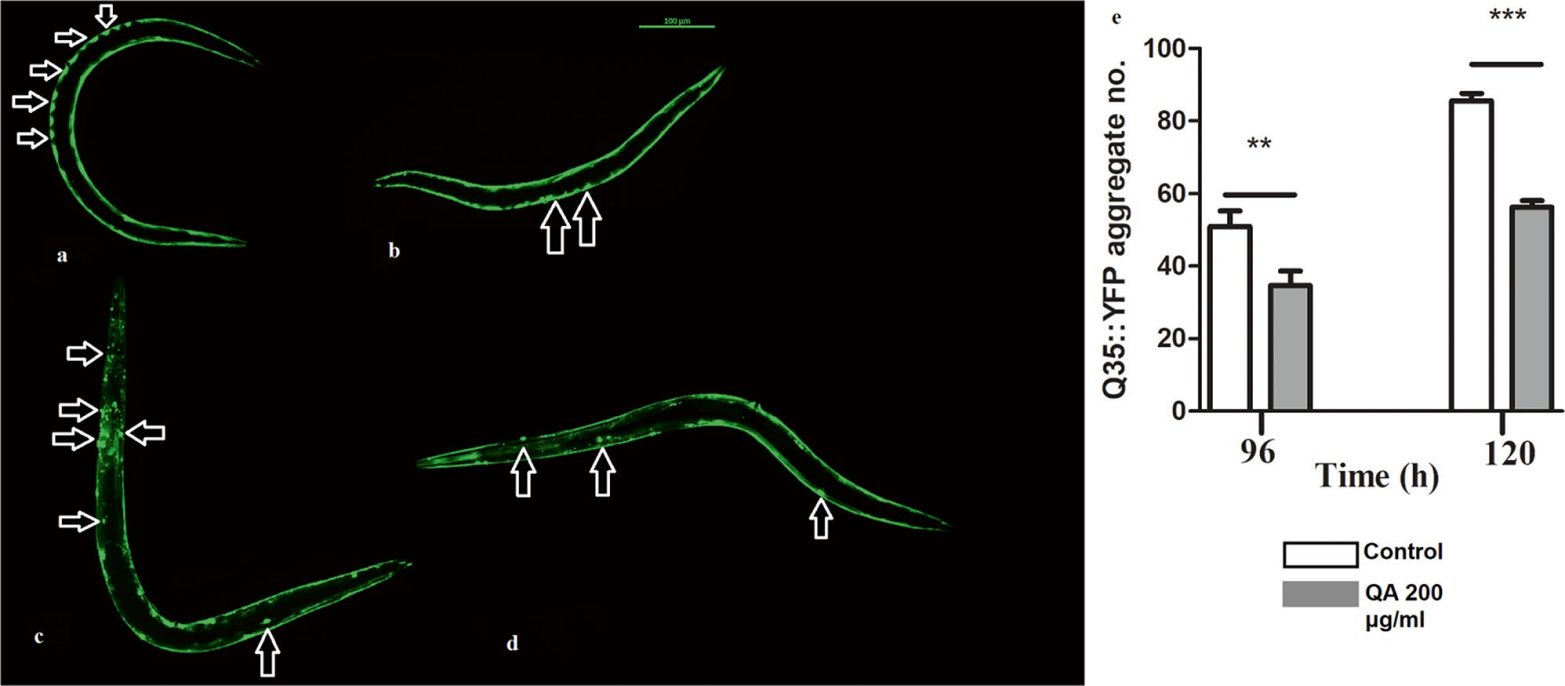
Effect of QA on polyQ35 aggregate formation in AM140 worms. **a** untreated control worm photographed after 96 h. **b** QA 200 µg/ml worm photographed after 96 h. **c** untreated control worm photographed after 120 h. **d** QA 200 µg/ml worm photographed after 120 h. Scale bar = 100 μm. **e** QA 200 µg/ml resulted in decrease in polyQ35 aggregate formation after 96 and 120 h by 47% and 52% respectively in comparison to untreated control (**p < 0.01, ***p < 0.001). Results are represented as mean aggregate numbers ± SEM and data comparison was performed using Student’s t-test

We also investigated if QA had the potential to decrease protein aggregation utilizing AM140 and AM141 strains. AM140 and AM141 express Q35 and Q40 polyglutamine repeats, respectively, in the muscles of worms as they age. QA treatment (200 µg/ml) could noticeably decrease aggregate formation in AM140 worms after 96 h and 120 h by 47% and 52% respectively (**p < 0.01, ***p < 0.001) (Fig. 7).

**Fig. 8:**
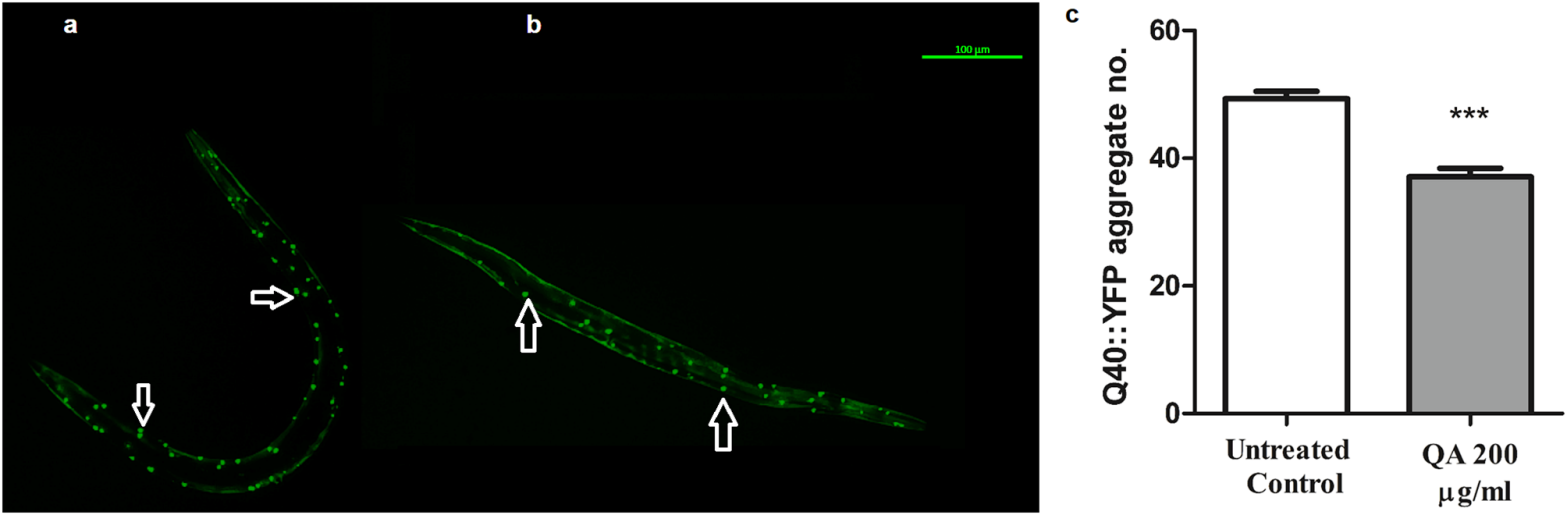
Effect of QA on polyQ40 aggregate numbers in AM141 worms. **a** untreated control worm. **b** QA 200 µg/ml worm. Scale bar = 100 μm. **c** QA 200 µg/ml resulted in decrease in polyQ40 aggregate formation by 33% compared with untreated control (***p < 0.001). Results are expressed as mean aggregate numbers ± SEM and data comparison was performed using Student’s t-test

Moreover, QA-treated worms exhibited a significant decline in polyQ40 aggregate numbers by 33% (***p < 0.001) (Fig. 8).

### QA increased body bending in AM141 and AM140 worms

**Fig. 9:**
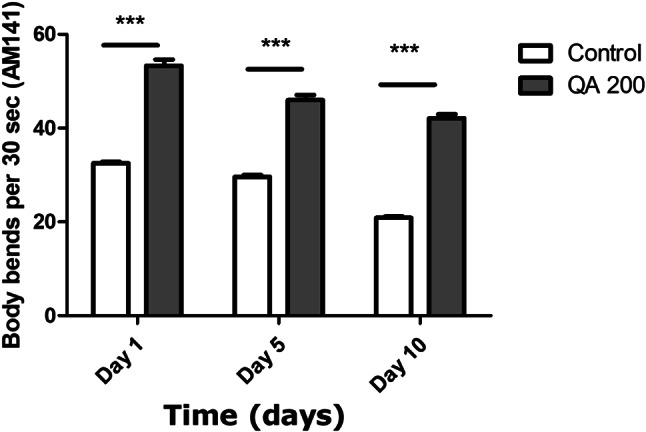
Body bending assay in AM141 worms. QA 200 µg/ml resulted in an increase in the number of body bends after 1, 5 and 10 d by 64%, 55% and 101% respectively in comparison to the untreated control group (***p < 0.001). Results are shown as mean numbers of body bends per 30 s ± SEM and data comparison was performed using Student’s t-test

Body bending is considered to be one of the markers of aging as it usually declines over time. In AM141 and AM140 worms, aging leads to an increase in the formation of polyQ40 and polyQ35 aggregates which decreases the body bending ability of worms. In AM141worms treated with 200 µg/ml QA, there was a significant raise in bending rate after day 1, 5 and 10 of adulthood by 64%, 55% and 101% respectively compared to the untreated control worms (***p < 0.001) (Fig. 9).

**Fig. 10:**
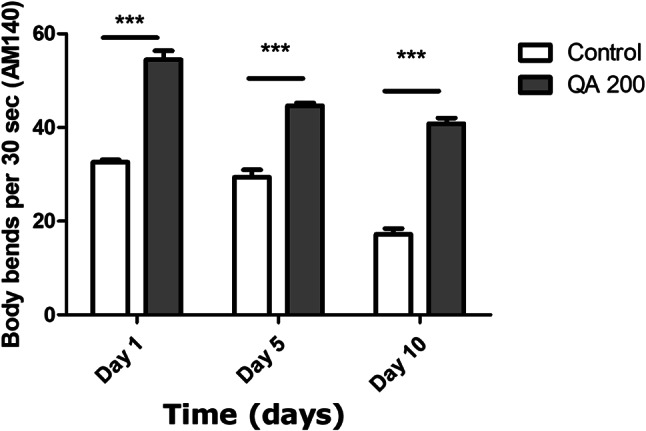
Body bending assay in AM140 worms. QA 200 µg/ml resulted in an increase in the number of body bends after 1, 5 and 10 d by 67%, 52% and 137% respectively in comparison to the untreated control group (***p < 0.001). Results are shown as mean numbers of body bends per 30 s ± SEM and data comparison was performed using Student’s t-test

In AM140 worms, treated with 200 µg/ml QA, there was a significant raise in bending rate after day 1, 5 and 10 of adulthood by 67%, 52% and 137% respectively compared to the untreated control worms (***p < 0.001) (Fig. 10).

## Discussion

Since ROS play critical roles in the process of aging and its associated disorders, antioxidants are very important in protecting our bodies through detoxifying excess ROS. Our bodies’ inherent antioxidant defense mechanisms are insufficient to counteract the high levels of ROS so people nowadays depend on synthetic antioxidants that are sometimes not very safe to use. This was a motive for scientists to start searching for natural sources containing antioxidant compounds that can be used more safely. Since plants represent a wide source of compounds with antioxidant activities, it is worth studying the natural compounds present in them [1, 7, 8, 22, 44].

Several neurodegenerative disorders are characterized by the formation of protein insoluble and toxic aggregates. HD is considered as a polyglutamine disorder because it is developed due to the formation of 35 or more CAG repeats on Htn protein forming polyQ oligomers and aggregates [45–48].

HD development correlates with oxidative damage so compounds with antioxidant potential might be helpful in preventing the development of the disease [49]. ROS can cause direct neuronal damage and contribute to the pathogenic cascade of HD [50]. In this study, we tested the antioxidant potential and protective activity of QA against the development of polyQ aggregates through using the *C. elegans* worm model.

*C. elegans* worms are particularly useful in studying neurodegenerative disorders like HD because, despite lacking strains that express a Huntingtin protein orthologue, there are several transgenic strains that express certain reporters which makes it easier to examine the expression and localization of specific proteins. Strains used for studying HD show neurodegeneration and mechanosensory defects by aging and increasing the length of polyQ tract [22, 51–53].

The two main highly conserved transcription factors responsible for controlling longevity and stress resistance in *C. elegans* are: SKN-1 and DAF-16 [22, 54]. DAF-16 is homologous to FOXO in humans and is responsible for regulating the expression of several genes that control lifespan through affecting stress resistance and longevity [55–57]. Upregulating SKN-1 increases the expression of cytoprotective genes resulting in better maintenance of cellular redox balance, increase in lifespan and resistance against pro-oxidants. On the contrary, when it is disrupted, aging is accelerated [25]. SKN-1 is an orthologue to Nrf2 and it was reported that Nrf2 activation exhibited protective effects in a mouse model of HD by decreasing motor disabilities, increasing life span and protecting neurons [58]. It was also previously reported that proteasome induction upon exposure to oxidative stress depends on Nrf2, a mechanism conserved for SKN-1 in *C. elegans*. This encouraged us to explore whether our drug affects SKN-1 expression and if it affects the formation of different types of polyQ aggregates, related to HD [59].

A former study using *C. elegans* proved that QA was able to decrease ROS levels, increase worm survival after exposure to heat and oxidative stress conditions using juglone and increase the expression of DAF-16 with its downstream gene, *sod-3* [17]. In our current study, QA demonstrated increased survival of worms exposed to H_2_O_2_ in N2, *skn-1* and *daf-16* null mutants. This result proves that DAF-16 is not the sole contributor in the activity of QA and that SKN-1 might be involved. Furthermore, our study indicated that QA can activate the Ld-1 SKN-1 pathway, which was confirmed by increased *skn-1* nuclear localization while also increasing the expression of its downstream Phase II detoxification enzymes GCS-1 and GST-4. This result confirmed the involvement of Ld-1 SKN-1 pathway in the oxidative stress resistance activity mediated by QA.

It is also interesting to note that caffeoylquinic acid, also present in our normal daily diet in coffee, tea and fruits, could increase the lifespan of *C. elegans*, postpone the age-related reduction in body movement, and enhance the worms’ resistance to both oxidative and heat stress. Those effects are believed to be mediated through DAF-16, SKN-1, HSF-1 and HIF-1 [60, 61].

Previous research had suggested that QA provides neuroprotective activities; this has been demonstrated by improvement in behavioral properties of memory and protection against dementia induced via aluminum chloride in rats [18]. Herein, by studying the effect of QA on *C. elegans* HD model, we determined through neuronal viability and chemotaxis assays that QA reduced ASH neuronal death, which is caused by polyQ150 aggregate formation. It was also able to decrease polyQ35 and polyQ40 formation in muscles, which improved body bending abilities of the worms.

## Conclusions

The current study provides an insight into the neuroprotective ability of QA against HD in the *C. elegans* worm model. QA has shown antioxidant properties through its ability to activate SKN-1/Nrf2 pathway and its downstream target genes *gcs-1* and *gst-4*. It exerted oxidative stress resistance activity by increasing the survival in wild type, *skn-1* and *daf-16* null mutant worms subjected to H_2_O_2_. We conclude that the protective ability of QA against HD is achieved by decreasing the accumulation of polyQ150, polyQ35 and polyQ40 aggregates. This effect could be partially modulated by the activation of SKN-1 pathway. However, other unidentified mechanisms can not be ruled out. Further studies are still required to show the specific molecular mechanisms affecting QA activity.

## Electronic supplementary material

Below is the link to the electronic supplementary material.

Supplementary Material 1

## Data Availability

Data of the current study will be available upon reasonable request from the corresponding author. Contact Dr. Sara Thabit: sara.thabit@guc.edu.eg.

## Abbreviations

ASH: Amphid sensilla
*C. elegans*: Caenorhabditis elegans
*E. coli*: Escherichia coli
FUDR: 5′-Fluorodeoxyuridine
GCS-1: Gamma-glutamyl cysteine synthetase
GFP: Green fluorescent protein
GST-4: Glutathione S-transferase-4
HD: Huntington’s disease
Htn: Huntingtin
H_2_O_2_: Hydrogen peroxide
NGM: Nematode growth medium
Nrf2: Nuclear factor erythroid 2-related factor 2
PolyQ: Polyglutamine
QA: Quinic acid
ROS: Reactive oxygen species
SKN-1: SKiNhead-1
