# Supplementary material for: Quinic acid protects against the development of Huntington’s disease in Caenorhabditis elegans model
Source: BMC Complement Med Ther. 2024 Oct 28;24:377. doi: 10.1186/s12906-024-04670-4 (PMC11514749; doi:10.1186/s12906-024-04670-4)
Supplement: Supplementary file 1 — Supplementary Material 1 [file 12906_2024_4670_MOESM1_ESM.pdf]

Quinic acid protects against the development of Huntington's disease in *Caenorhabditis elegans* model

Reem Hossam El Din[1], Sara Thabit[2*]

[1]Department of Pharmaceutical Microbiology, Faculty of Pharmacy and Biotechnology, German University in Cairo, New Cairo, Egypt

[2]Department of Pharmaceutical Biology, Faculty of Pharmacy and Biotechnology, German University in Cairo, New Cairo, Egypt

Correspondence: Dr. Sara Thabit, Faculty of Pharmacy and Biotechnology, Pharmaceutical Biology Department, German University in Cairo, New Cairo 11835, Egypt. E. mail: sara.thabit@guc.edu.eg

Figure.S1

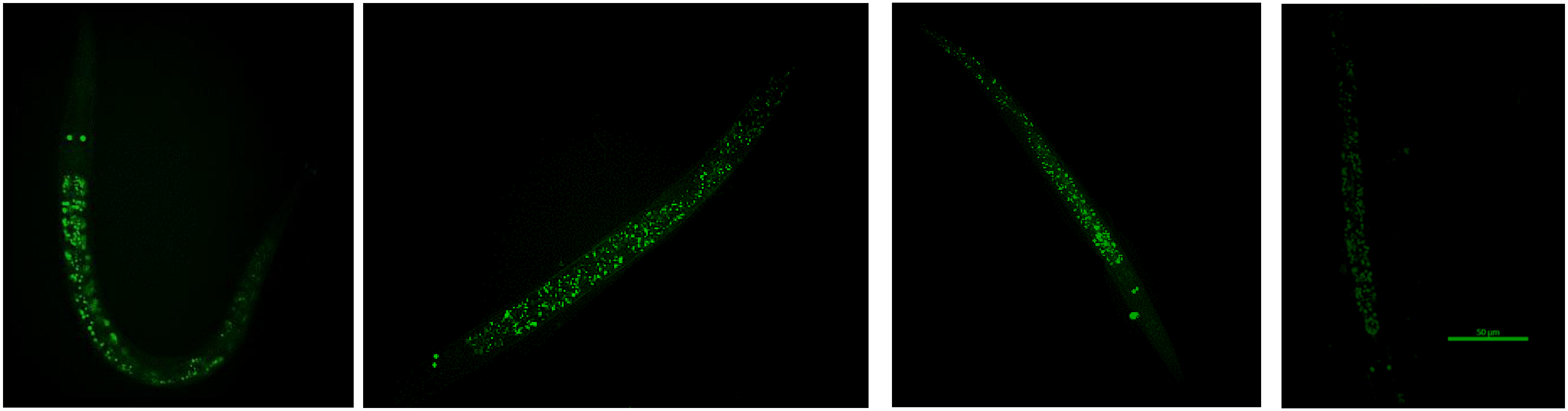

(a) untreated control (b) QA 50 µg/ml (c) QA 100 µg/ml (d) QA 200 µg/ml

Nuclear localization

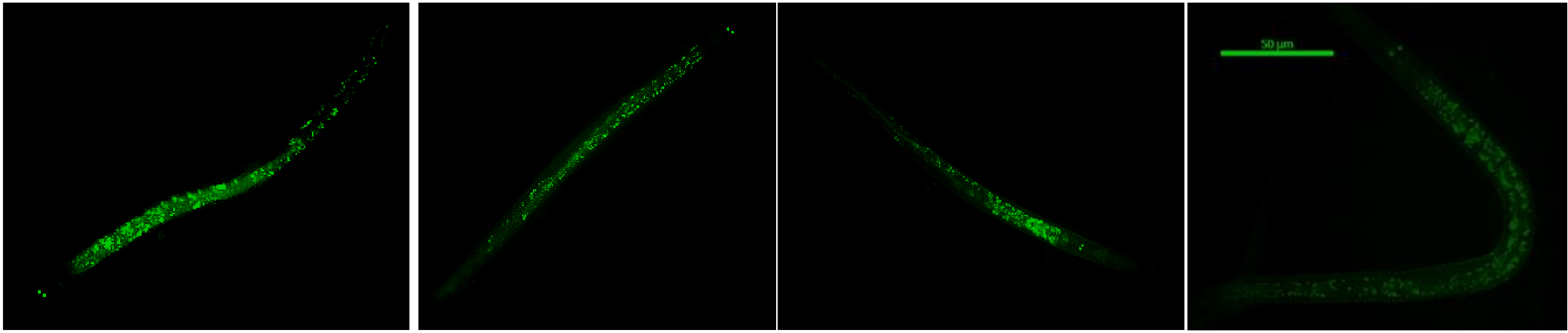

(e) untreated control (f) QA 50 µg/ml (g) QA 100 µg/ml (h) QA 200 µg/ml

Cytoplasmic localization

Supplementary figure S1:

Micrographs of worms showing Ld-1 SKN-1 localization. Scale bar = 50 µm.

Figure.S2

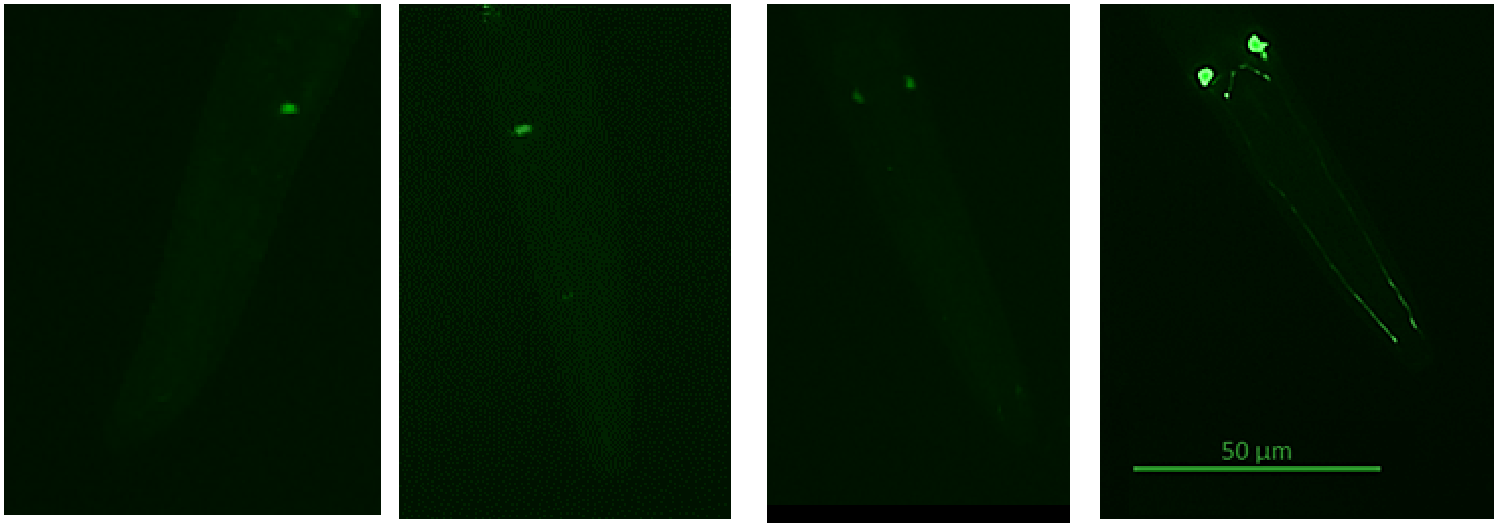

a b c d

Supplementary figure S2:

Micrographs of worms showing ASH neuronal survival. (a) HA759 worm showing death in one of the ASH neurons in control group. (b) HA759 worm showing death in one of the ASH neurons in QA 200 µg/ml group. (c) HA759 worm with bilateral ASH neurons in control group. (d) HA759 worm with bilateral ASH neurons in QA 200 µg/ml group. Scale bar = 50 µm.

Figure.S3

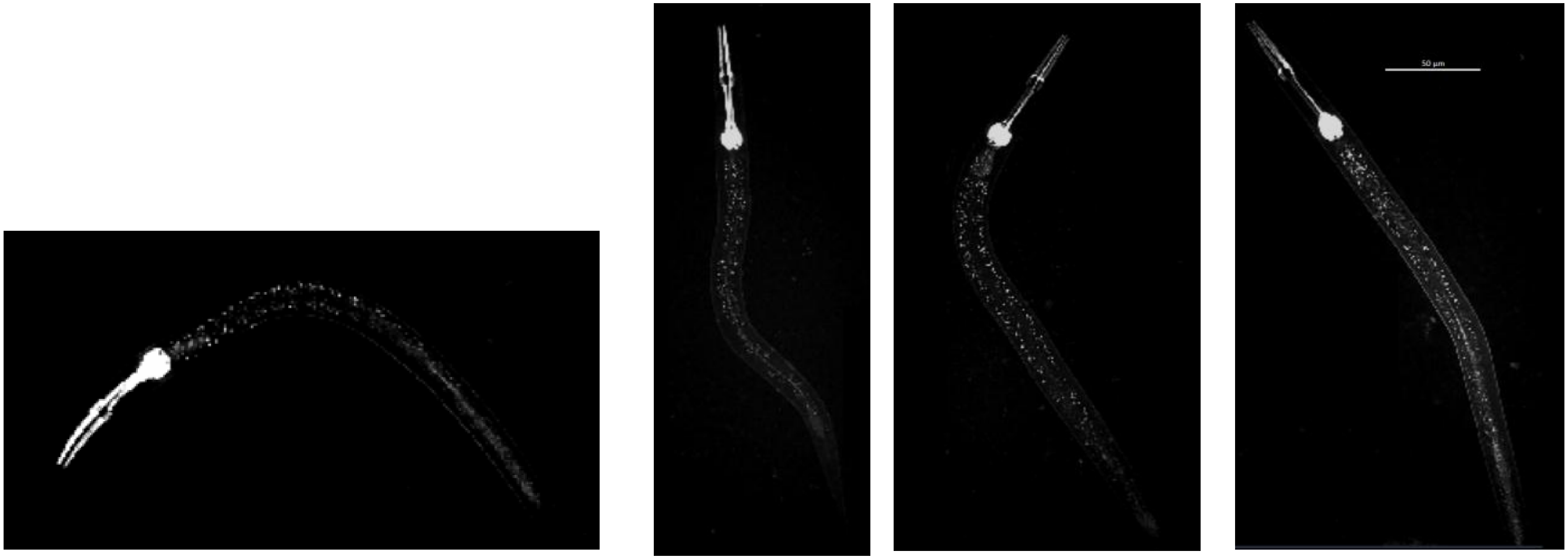

(a) untreated control (b) QA 50 µg/ml (c) QA 100 µg/ml (d) QA 200 µg/ml

Supplementary figure S3:

Micrographs of worms showing expression of GCS-1. Scale bar = 50 µm.

Figure.S4

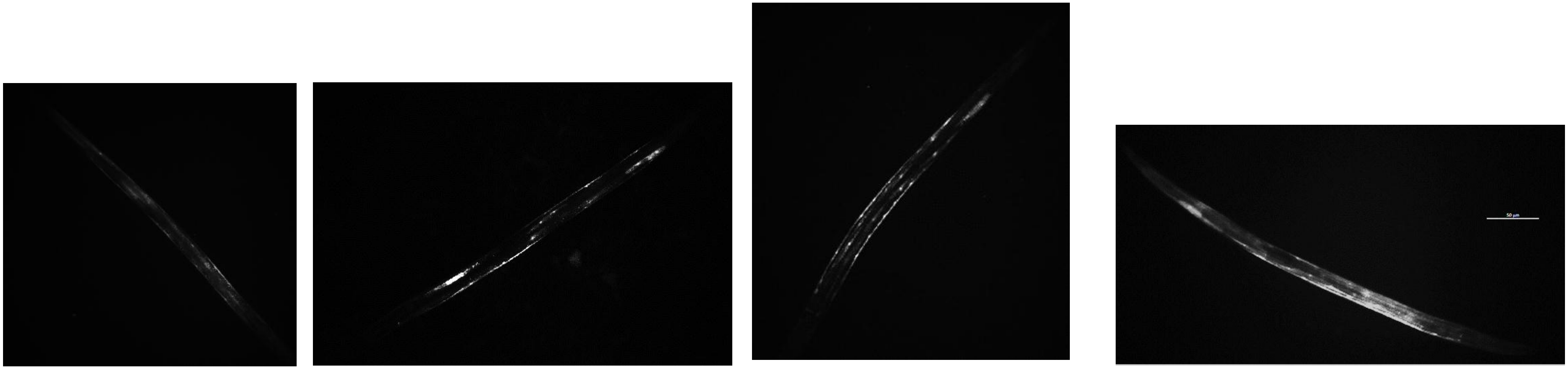

(a) untreated control (b) QA 50 µg/ml (c) QA 100 µg/ml (d) QA 200 µg/ml

Supplementary figure S4:

Micrographs of worms showing expression of GST-4. Scale bar = 50 µm.

Figure.S5

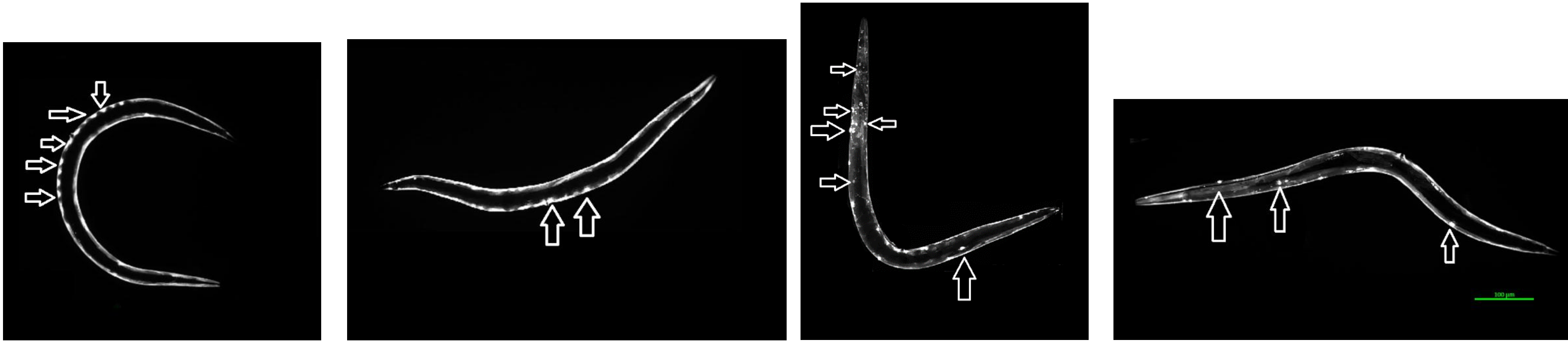

(a) untreated control after 96 h

(b) QA 200 µg/ml after 96 h

(c) untreated control after 120 h

(d) QA 200 µg/ml after 120 h

Supplementary figure S5:

Micrographs of worms showing polyQ35 aggregate formation in AM140 worms. Scale bar = 100 µm.

Figure.S6

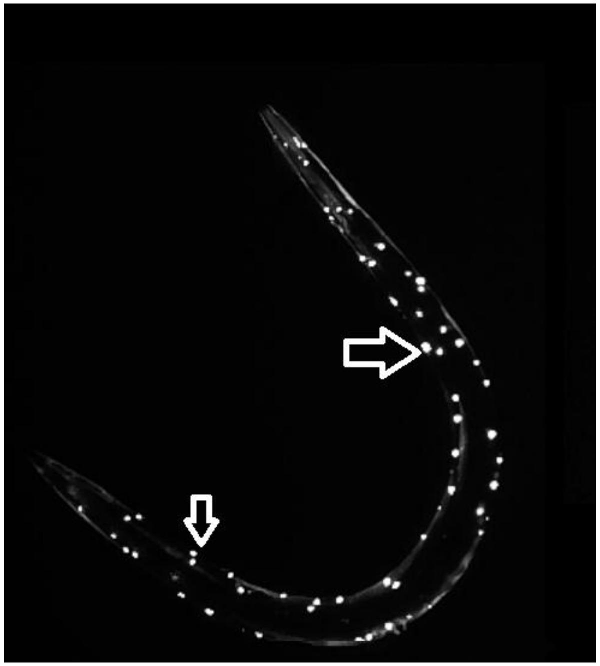

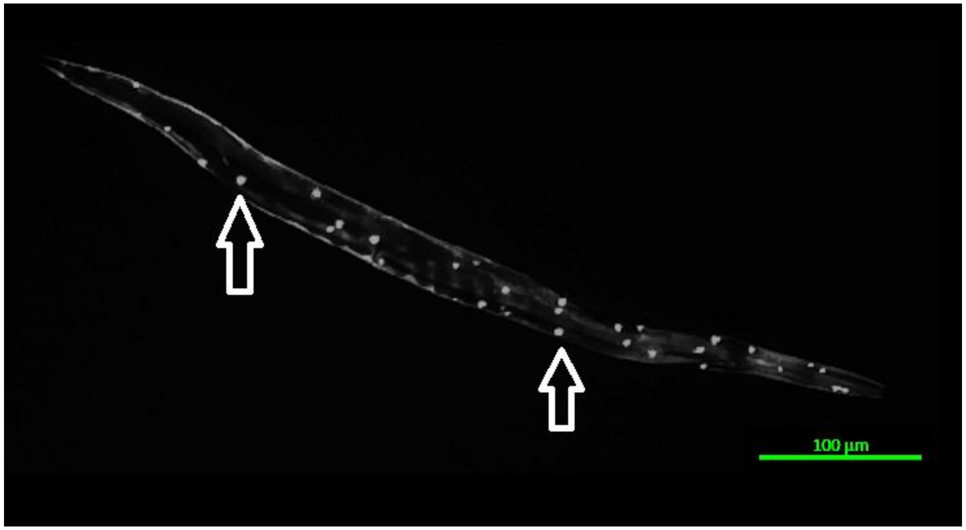

(a) untreated control

(b) QA 200 µg/ml

Supplementary figure S6:

Micrographs of worms showing polyQ40 aggregates in AM141 worms. Scale bar = 100 µm.
